# Special Issue “miRNAs in the Era of Personalized Medicine: From Biomarkers to Therapeutics 2.0”

**DOI:** 10.3390/ijms24031951

**Published:** 2023-01-19

**Authors:** Bárbara Andrea Mc Cormack, Eva González-Cantó, Sarai Tomás-Pérez, Cristina Aghababyan, Josep Marí-Alexandre, Martin Götte, Juan Gilabert-Estellés

**Affiliations:** 1 Research Laboratory in Biomarkers in Reproduction, Gynaecology and Obstetrics, Research Foundation of the General University Hospital of Valencia, 46014 Valencia, Spain; 2 Department of Obstetrics and Gynaecology, General University Hospital of Valencia Consortium, 46014 Valencia, Spain; 3 Department of Pathology, General University Hospital of Valencia Consortium, 46014 Valencia, Spain; 4 Research Laboratory, Department of Gynecology and Obstetrics, Münster University Hospital, 48149 Münster, Germany; 5 Department of Pediatrics, Obstetrics and Gynaecology, University of Valencia, 46010 Valencia, Spain

Personalized medicine has become a new paradigm in the management of a variety of diseases [1]. Thus, the interest in deepening the knowledge of an individual molecular characterization of pathological entities has spread from medical specialists to scientific research groups. In recent years, technological advancements have allowed the development of risk stratification algorithms, biomarker analysis and targeted therapies. Although a limited number of techniques have been incorporated in routine clinical practice, there is no doubt that their use in research laboratories contributes to improving the understanding of diseases and the search for more appropriate therapies [2].

In the last decade, the advantage that poses the stability and tissue specificity of the non-coding miRNAs (ncRNAs) has postulated them as a candent area of research [3]. Among ncRNAs, microRNAs (miRNAs) represent the most abundant subgroup and have attracted particular attention since they have proven to be deregulated in several pathologies, from benign ones, such as endometriosis or cardiovascular diseases, to distinct forms of solid and hematological malignancies [3,4,5]. Additionally, since miRNAs could impact multiple signaling pathways involved in the pathophysiology of various diseases, they have become potentially valuable therapeutic targets. Moreover, the discovery of miRNAs in a myriad of human biofluids paved the way for their implementation as biomarkers of diseases [4].

Undoubtedly, the miRNA-personalized medicine couple is a topic of great interest and constant enrichment [6]. To encompass the novel findings in this area, the Special Issue "miRNAs in the Era of Personalized Medicine: From Biomarkers to Therapeutics” has been reopened in its updated 2.0 version. Since miRNAs are involved in a wide range of pathologies, the current Special Issue intends to cover advancements in those diseases not mentioned in the previous edition, as well as deepen our knowledge in those pathologies already mentioned, such as Glioblastoma and skin diseases. As a result, five original manuscripts and four review articles have been compiled, covering not only tumor pathologies but also inflammatory and chronic diseases, radiation exposure effect, and basic research on the miRNAs mechanism of action, as summarized in Table 1.

According to the Special Issue title, there are at least three starting points for miRNA research in personalized medicine. Great effort is being made to elucidate its role as either a prognostic or diagnostic biomarker, to define its implication in the pathophysiology of several conditions, and finally, to increase knowledge about its potential as a therapeutic target or therapeutic agent.

With regard to the role of miRNAs as biomarkers, the authors of “Circulating tRNA-Derived Small RNAs as Novel Radiation Biomarkers of Heavy Ion, Proton and X-ray Exposure” [7] developed a multi-factor model based on tRNA-derived small RNA biomarkers to indicate the degree of radiation exposure with high sensitivity and specificity by a total-body exposure of mice to carbon ions assay with its corresponding human validation.

Focusing on the role of miRNAs in the diagnosis and prognosis of pathologies, the authors of “miR-10 and Its Negative Correlation with Serum IL-35 Concentration and Positive Correlation with STAT5a Expression in Patients with Rheumatoid Arthritis” [8] aimed to find a possible association between 13 selected serum-miRNAs, Th17/Treg transcription factor expression, and clinical features in patients with rheumatoid arthritis (RA). Experiments performed on patients with RA and osteoarthritis (OA), as well as healthy subjects (HC) showed a differential association between transcription factor expression and serum miRNA levels that could be relevant for the diagnosis and progression analysis of RA and OA.

Identifying those aspects that characterize pathology is also a way to deepen our knowledge. To identify miRNAs and pathways specifically deregulated in adolescent and young adult T-cell acute lymphoblastic leukemia patients, Dawidoska et.al. [9] analyzed the miRNA expression by RNA-seq. A downregulation of miR-143-3p was observed and its putative involvement in the biology of leukemia was evaluated by in silico predictions and basic functional experiments. This research demonstrates how a detailed definition of the molecular landscape of a disease could lead to an improvement in its treatment through the identification of specific therapeutic features.

Regarding the therapeutic axis, there are many steps to be undertaken in the search for a new therapy. Firstly, it is essential to dispose of experimental techniques capable of detecting and functionally evaluating the events or targets of interest. In this regard, the authors of “Functional Screen for microRNAs Suppressing Anchorage-Independent Growth in Human Cervical Cancer Cell” [10] successfully developed alternative functional screening methods to systematically investigate the microRNA regulation of anchorage-independent growth in cervical cancer cells. Moreover, the validation of this screening in three cervical carcinoma cell lines yielded 40 microRNAs that specifically reduced anchorage-independent growth in this disease. This practical knowledge might provide tools for further functional screenings.

Secondly, defining the therapeutic target is also a critical event in the development of a new treatment or drug. In this sense, the authors of “The Increase of miR-195-5p Reduces Intestinal Permeability in Ulcerative Colitis, Modulating Tight Junctions’ Expression” [11] carried out a comprehensive characterization of the distribution of tight junctions and alteration of barrier permeability in inflammatory bowel disease, both in vitro and in vivo, and were able to point out to a specific miRNA (miR-195-5p) involved in those processes. These results give evidence of a potential miRNA-based therapy for inflammatory bowel disease.

Focused on developing new therapeutic approaches based on selective knockdown of broadly abnormally expressed miRNAs, Patutina et al. performed an innovative study entitled “Bulge-Forming miRNases Cleave Oncogenic miRNAs at the Central Loop Region in a Sequence-Specific Manner” [12]. The authors engineered a series of bulge-loop-forming oligonucleotides conjugated with catalytic peptides, capable of recognizing and destroying the oncogenic-miRNAs, thus achieving a desired therapeutic effect. Accordingly, the authors of "Synergistic Effects of A Combined Treatment of Glioblastoma U251 Cells with An Anti-miR-10b-5p Molecule and An Anti-Cancer Agent Based on 1-(3′,4′,5′-Trimethoxyphenyl)-2-Aryl-1H-Imidazole Scaffold" [13] support the idea that a combined cancer treatment involving molecules targeting specifically up-regulated miRNAs and an anticancer agent may represent a promising strategy, as it involves a personalized approach.

In addition to these original papers, four review articles have been included in this Special Issue. They present several up-to-date aspects of the role of miRNAs in different pathologies: inflammatory skin conditions, idiopathic pulmonary fibrosis, pituitary neuroendocrine tumors, and lung cancer.

Specifically, Borgia et al. [14] summarized the knowledge on the role of miRNAs in acne vulgaris and hidradenitis suppurativa, two inflammatory pathologies that often coexist. The authors aimed to identify possible commonly deregulated miRNAs that could explain the similar characteristics of these two diseases and set a starting point for further research looking for a common miRNA-based treatment.

Cadena-Suárez et al. [15] produced a thoughtful review by analyzing and discussing the putative association of miRNAs with the signaling pathways involved in the development of idiopathic pulmonary fibrosis. The authors based their review on works that assessed the signaling pathways involved with epithelial–mesenchymal transition, the fibroblast differentiation, and synthesis of extracellular matrix components.

Butz [16] reviewed the available data on circulating ncRNAs as a potential diagnostic and prognostic biomarker in pituitary neuroendocrine tumors. Authors highlight the existence of discrepancies, which could explain why their use as biomarkers is currently not feasible in clinical laboratories.

Konoshenko et al. [17] compile and discuss available data in the literature and bioinformatic resources on the expression of miRNAs as potential predictive markers of drug resistance in lung cancer. This review presents an overview of published data and highlights the potential of miRNAs as a perspective tool for the cisplatin therapy’s efficacy evaluation and prediction. 

In conclusion, the articles presented in this updated Special Issue represent scientific efforts to understand the role of miRNAs in the mechanism of disease, their potential employment as diagnostic and prognostic biomarkers, and their putative use as therapeutic targets. As our knowledge is deepened and shared with the scientific and healthcare community, we will hopefully move closer to establishing a more personalized medical treatment system.

As guests editors, we would like to acknowledge all the authors and research groups for their participation in the Special Issue, as well as the staff members of the International Journal of Molecular Science (IJMS) for their editorial support.

## Figures and Tables

**Table 1:** Contributors to the Special Issue.

Authors	Title	Topic	Type
Wei et.al. [7]	Circulating tRNA-Derived Small RNAs as Novel Radiation Biomarkers of Heavy Ion, Proton and X-ray Exposure.	Radiation Biomarkers	Original Research
Paradowska-Gorycka et.al. [8]	miR-10 and Its Negative Correlation with Serum IL-35 Concentration and Positive Correlation with STAT5a Expression in Patients with Rheumatoid Arthritis	Rheumatoid Arthritis	Original Research
Dawidoska et.al. [9]	Small RNA-Seq Reveals Similar miRNA Transcriptome in Children and Young Adults with T-ALL and Indicates miR-143-3p as Novel Candidate Tumor Suppressor in This Leukemia	Leukemia	Original Research
Huseinovic et.al. [10]	Functional Screen for microRNAs Suppressing Anchorage-Independent Growth in Human Cervical Cancer Cells.	Cervical Cancer	Original Research
Scalavino et.al. [11]	The Increase of miR-195-5p Reduces Intestinal Permeability in Ulcerative Colitis, Modulating Tight Junctions’ Expression.	Ulcerative Colitis	Original Research
Patutina et.al. [12]	Bulge-Forming miRNases Cleave Oncogenic miRNAs at the Central Loop Region in a Sequence-Specific Manner.	Cancer-associated miRNAs mechanisms	Original Research
Zurlo et.al. [13]	Synergistic Effects of A Combined Treatment of Glioblastoma U251 Cells with An Anti-miR-10b-5p Molecule and An Anti-Cancer Agent Based on 1-(3′,4′,5′-Trimethoxyphenyl)-2-Aryl-1H-Imidazole Scaffold	Glioblastoma	Original Research
Borgia et al. [14]	MicroRNA Cross-Involvement in Acne Vulgaris and Hidradenitis Suppurativa: A Literature Review	Inflammatory skin conditions	Review
Cadena-Suárez et.al. [15]	Role of MicroRNAs in Signaling Pathways Associated with the Pathogenesis of Idiopathic Pulmonary Fibrosis: A Focus on Epithelial-Mesenchymal Transition.	Pulmonary Fibrosis	Review
Butz. [16]	Circulating Noncoding RNAs in Pituitary Neuroendocrine Tumors-Two Sides of the Same Coin.	Neuroendocrine Tumors	Review
Konoshenko et al. [17]	MicroRNAs as Predictors of Lung-Cancer Resistance and Sensitivity to Cisplatin	Lung Cancer	Review
